# SUNny Ways: The Role of the SUN-Domain Protein Mps3 Bridging Yeast Nuclear Organization and Lipid Homeostasis

**DOI:** 10.3389/fgene.2020.00136

**Published:** 2020-02-28

**Authors:** Maria Laura Sosa Ponce, Sarah Moradi-Fard, Vanina Zaremberg, Jennifer A. Cobb

**Affiliations:** ^1^ Departments of Biochemistry & Molecular Biology and Oncology, Robson DNA Science Centre, Arnie Charbonneau Cancer Institute, Cumming School of Medicine, Calgary, AB, Canada; ^2^ Department of Biological Sciences, University of Calgary, Calgary, AB, Canada

**Keywords:** telomeres, lipid metabolism, transcription, nuclear envelope, SUN-domain proteins

## Abstract

Mps3 is a SUN (Sad1-UNC-84) domain-containing protein that is located in the inner nuclear membrane (INM). Genetic screens with multiple Mps3 mutants have suggested that distinct regions of Mps3 function in relative isolation and underscore the broad involvement of Mps3 in multiple pathways including mitotic spindle formation, telomere maintenance, and lipid metabolism. These pathways have largely been characterized in isolation, without a holistic consideration for how key regulatory events within one pathway might impinge on other aspects of biology at the nuclear membrane. Mps3 is uniquely positioned to function in these multiple pathways as its N- terminus is in the nucleoplasm, where it is important for telomere anchoring at the nuclear periphery, and its C-terminus is in the lumen, where it has links with lipid metabolic processes. Emerging work suggests that the role of Mps3 in nuclear organization and lipid homeostasis are not independent, but more connected. For example, a failure in regulating Mps3 levels through the cell cycle leads to nuclear morphological abnormalities and loss of viability, suggesting a link between the N-terminal domain of Mps3 and nuclear envelope homeostasis. We will highlight work suggesting that Mps3 is pivotal factor in communicating events between the nucleus and the lipid bilayer.

*The Wind and the Sun (Æsop Fables (Sixth century B.C.). The Harvard Classics. 1909–14.*

THE WIND and the SUN were disputing which was the stronger. Suddenly they saw a traveller coming down the road, and the Sun said: “I see a way to decide our dispute. Whichever of us can cause that traveller to take off his cloak shall be regarded as the stronger. You begin.” So the Sun retired behind a cloud, and the Wind began to blow as hard as it could upon the traveller. But the harder he blew the more closely did the traveller wrap his cloak round him, till at last the Wind had to give up in despair. Then the Sun came out and shone in all his glory upon the traveller, who soon found it too hot to walk with his cloak on.

“KINDNESS EFFECTS MORE THAN SEVERITY.”

## MPS3 is a Structural Component of the Nuclear Envelope

The nucleus of a eukaryotic cell is demarcated by the nuclear envelope (NE), a double lipid bilayer structure composed of an inner nuclear membrane (INM) and an outer nuclear membrane (ONM). While the ONM is continuous with the endoplasmic reticulum (ER) and is very similar in protein and lipid composition, the composition of the INM is quite distinct (Schirmer and Gerace, 2005). The ONM and INM are joined throughout the NE by nuclear pore complexes (NPCs), which serve as gateways of transportation between the cytoplasm and nucleoplasm. The contribution of the NPC to NE structure has been reviewed extensively elsewhere and will not be discussed here (Knockenhauer and Schwartz, 2016; Beck and Hurt, 2017; Goldberg, 2017).

In higher eukaryotes, the structure of the nucleus is largely maintained by the nuclear lamina, a network of lamin proteins associated with the nucleoplasmic side of the INM (Shimi et al., 2010; Romero-Bueno et al., 2019) and Sad1-UNC-84 (SUN)-domain containing proteins first discovered from an ~150 amino-acid region of homology between Sad1 in *Schizosaccharomyces pombe* and UNC-84 in *Caenorhabditis elegans* (Hagan and Yanagida, 1995; Malone et al., 1999). SUN-domain containing proteins in higher eukaryotes interact with lamin and also contribute to NE structure by their involvement in the linker of nucleo-skeleton and cytoskeleton (LINC) complex. The LINC complex includes a SUN domain protein in the INM and a KASH (Klarsicht-Anc-1-Syne-1) domain protein in the ONM that interact with one another in the lumen of the NE (Crisp et al., 2006; Razafsky and Hodzic, 2009).

The SUN-domain containing protein in *Saccharomyces cerevisiae* is called Monopolar spindle 3 (Mps3). However, a canonical LINC complex has not been detected in budding yeast because a *bona fide* KASH domain-containing protein has not yet been identified (Friederichs et al., 2012). Csm4 was proposed to function as a ‘KASH’ partner during chromosome segregation in meiosis because it binds Mps3 and localizes to the ONM (Burri and Lithgow, 2004; Koszul and Kleckner, 2009; Morillo-Huesca et al., 2019). More recently, a Csm4 paralogue called Mps2 was identified as a KASH-like protein and shown to form a non-canonical SUN-KASH complex with Mps3 (Chen et al., 2019).

**Figure 1:**
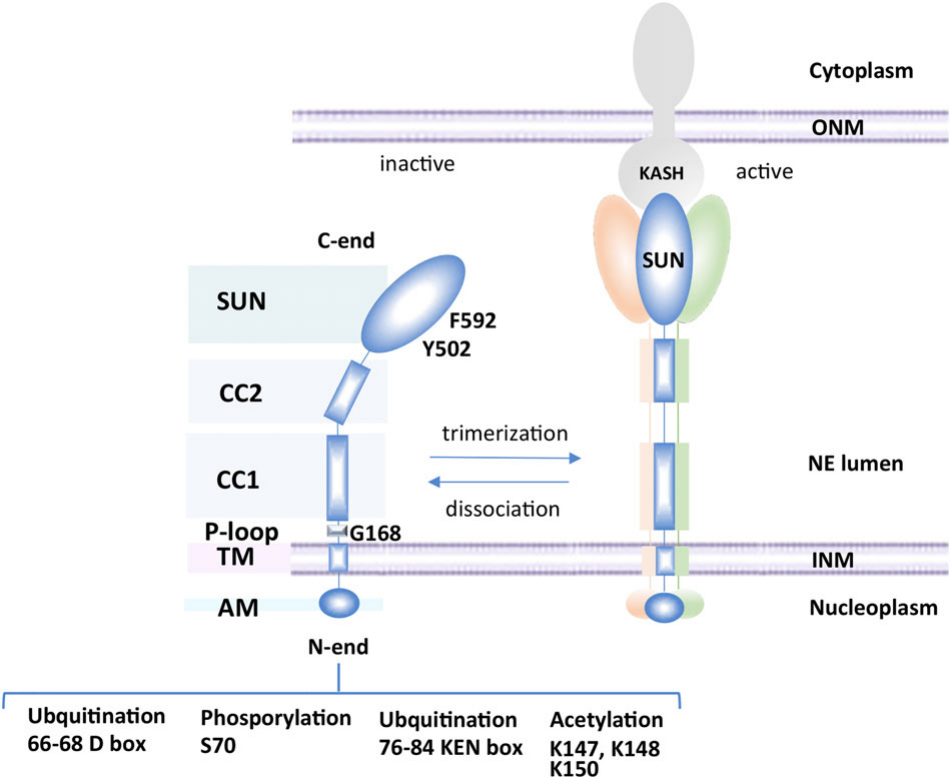
Schematic of Mps3 domains. Mps3 consists of an N-terminal nucleoplasmic region (1–150 aa), a transmembrane domain (154–181 aa), a P-loop (187–194 aa), two coiled-coil domains (242–260 and 366–390) and a SUN domain (427–616). SUN domains typically associate with KASH domain proteins in the outer nuclear membrane after trimerization of the SUN-domain protein. Mps3 can be modified by acetylation, ubiquitination or phosphorylation.

The structural organization of Mps3 is multifaceted (**Figure 1**). SUN-domain proteins typically form trimers that span the INM (Zhou et al., 2012; Nie et al., 2016). Consistently, Mps3 has been shown to oligomerize in yeast (Li et al., 2017). Each Mps3 monomer contains an N-terminal region (1–150 aa) extending into the nucleoplasm, a transmembrane domain (154–181 aa) spanning the INM, and many functional domains oriented within the perinuclear space, including an ATP binding P-loop (187–194 aa), coiled-coil domains (242–260 aa and 366–390 aa) and a SUN domain (427–616 aa) (Jaspersen et al., 2002; Jaspersen et al., 2006; Bupp et al., 2007). Based on work with mammalian Sun2, the SUN domain of Mps3 folds into a series of β-sheets (Sosa et al., 2012; Burke, 2018). Mutations in the SUN domain disrupt spindle pole body (SPB) organization, which is a major function of Mps3 in mitosis (Jaspersen et al., 2002; Nishikawa et al., 2003).

The cell cycle turnover of Mps3 is regulated by ubiquitination and degradation by Cdh1, which acts in late mitosis and early G1 (Koch et al., 2019). The regulated turnover of Mps3 depends on phosphorylation of S70, which is located between two anaphase-promoting complex (APC) destruction motifs in the N-terminal domain of Mps3, a KEN box (66–68 aa) and a D box (76–84 aa) (**Figure 1**) (Glotzer et al., 1991; Pfleger and Kirschner, 2000; Koch et al., 2019). The degradation of Mps3 in late mitosis likely contributes to spindle pole body disassembly. Failure to degrade Mps3 leads to its accumulation in the INM, aberrant nuclear envelope expansion, and an impairment in cell cycle progression (Friederichs et al., 2011; Li et al., 2017; Koch et al., 2019). Similarly, mutations in conserved residues in the SUN domain, like *mps3*-Y502H and *mps3*-F592S, show mitotic arrest as well as synthetic sickness or lethality in combination with the deletion of factors involved in lipid metabolism (Friederichs et al., 2012). Thus, two distinct regions of Mps3, which function in separate compartments, the N-terminal domain in the nucleoplasm and the SUN domain in the lumen, are implicated in mitotic progression and in NE proliferation. However, a genetic screen predicted that the distinct domains of Mps3 function in relative isolation, impacting pathways such as mitotic spindle formation, NPC insertion, chromatin organization, and lipid homeostasis (Friederichs et al., 2012). The role of Mps3 in SPB and NPC insertion has been reviewed elsewhere (Jaspersen and Ghosh, 2012) and there are multiple comprehensive reviews on the LINC complex and SUN-KASH interactions; we direct readers to these for details (Rothballer et al., 2013; Tapley and Starr, 2013; Hieda, 2017; Hao and Starr, 2019). In this mini-review, we will cover emerging evidence supporting a role for Mps3 in balancing lipid metabolism and NE homeostasis and links with telomere organization.

## MPS3 and Lipid Metabolism

Although no KASH proteins have been confirmed in *S. cerevisiae,* several tail-anchored proteins known to localize to the ER have been shown to physically interact with Mps3 and could equally be considered KASH-like partners (Burri and Lithgow, 2004; Bommi et al., 2019). One of these ONM proteins is Scs2, which has been linked to telomere silencing (Craven and Petes, 2001; Cuperus and Shore, 2002). Scs2 is a type II integral membrane protein, member of the VAP (VAMP/synaptobrevin-associated protein) family that localizes to the nuclear membrane, where it regulates phospholipid biosynthesis and lipid traffic (Loewen et al., 2003). Scs2 interacts with proteins containing FFAT motifs (two phenylalanines (FF) in an Acidic Tract). Among these is the transcriptional corepressor of phospholipid biosynthetic gene Opi1 (Loewen et al., 2003). The interaction between Scs2 and Opi1 favors binding of the transcriptional regulator to phosphatidic acid (PA) at the nuclear membrane and expression of lipid biosynthetic genes. Conditions that result in PA consumption favor the release of Opi1 from the ONM, allowing its translocation to the nucleus and subsequent repression of its target genes (Kliewe et al., 2017). Investigating a link between Scs2 and Mps3 could connect lipid homeostasis at the ONM with telomere silencing at the INM through known functions of Mps3, Scs2 or both.

**Figure 2:**
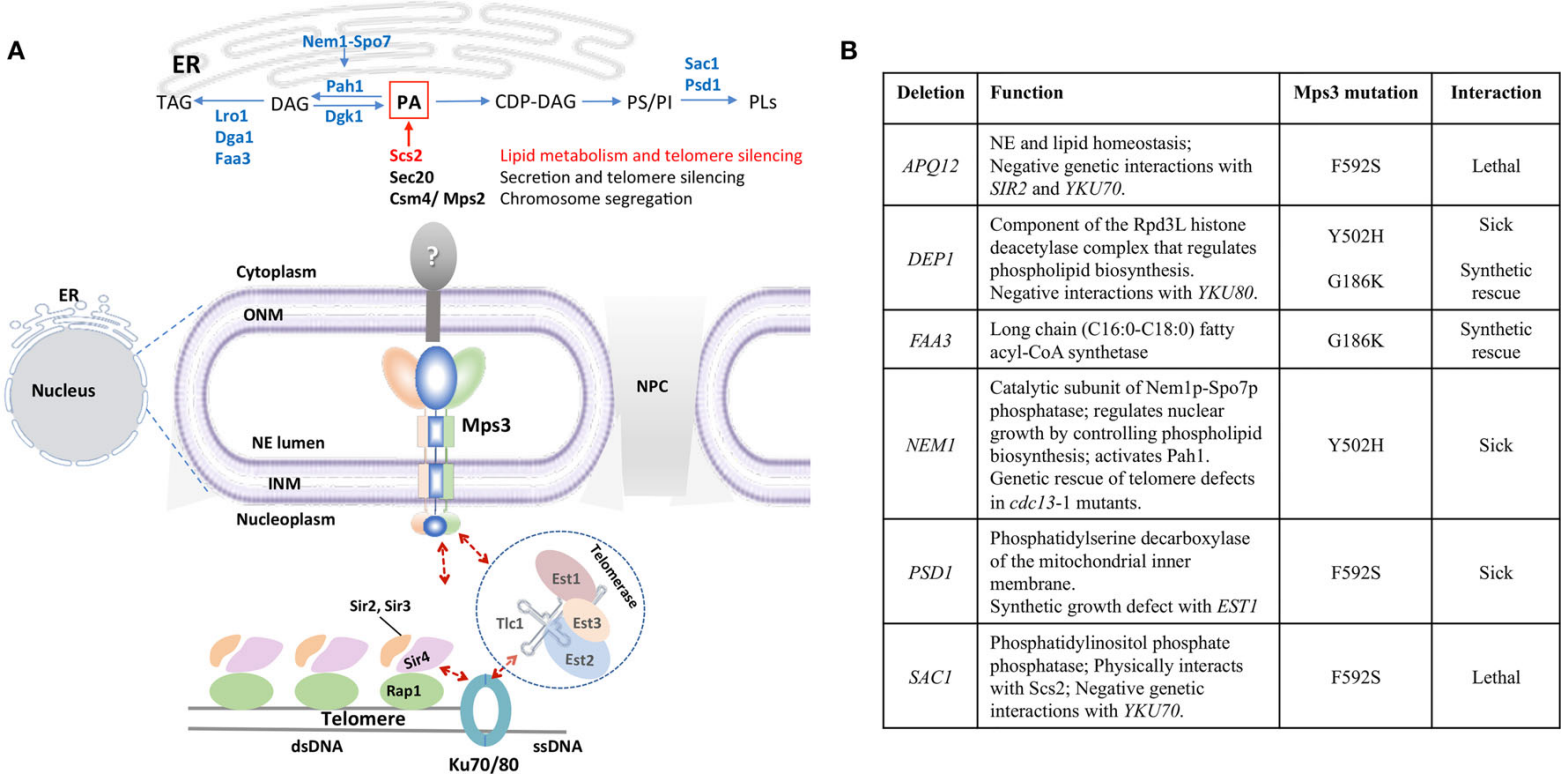
Mps3 is at the crossroads of lipid metabolism and telomere organization. **(A)** Mps3 participates in tethering of telomeres through interactions with Sir4 and Est1. These interactions facilitate maintenance of telomere length, telomere clustering, and telomere silencing. The position of Mps3 in the nuclear membrane makes it a potential sensor to communicate changes in membrane composition to the nucleus, potentially affecting telomere regulation. Potential KASH-like binding partners of Mps3 like Scs2 (red), Sec20, and Csm4/Mps2 are indicated, as well as enzymes related to lipid metabolism mentioned in the text. DAG, diacylglycerol; PA, phosphatidic acid; PI, phosphatidylinositol; PS, phosphatidylserine; PLs, phospholipids; TAG, triacylglycerol. **(B)** Summary of the genetic interactions between mps3 mutants and genes involved in lipid biosynthesis regulation.

Considering the potential for Mps3 to affect lipid homeostasis through Scs2, it is interesting to note that several Mps3 mutants have been shown to affect lipid levels (Friederichs et al., 2011; Ohsaki et al., 2016). In order to understand the effect of these mutants on nuclear structure, one must have a concept of the lipid metabolic pathway. In brief, PA is the precursor for all glycerolipids, and represents a branching point between membrane synthesis and energy storage pathways. In yeast, conversion of PA to CDP-diacylglycerol (CDP-DAG) channels metabolism towards phospholipid biosynthesis (**Figure 2A**). Conversion of PA to DAG by the PA phosphatase Pah1 diverts the metabolic pathway towards the synthesis of the storage lipid triacylglycerol (TAG), based on the cellular demand for energy storage during cessation of growth [**Figure 2A**; reviewed in (Siniossoglou, 2013)]. Synthesis of TAG leads to the emergence of lipid droplets (LDs), which are micellar organelles that store and metabolize neutral lipids (Walther et al., 2017). LDs serve as energy reservoirs that are consumed during resumption of growth.

It has been postulated that maintaining a balance between DAG and PA levels is important for the maintenance of NE structure (Barbosa et al., 2015). This has been shown in cells that undergo mitotic arrest or accumulate PA, which develop an extension of the NE known as a “nuclear flare” (Campbell et al., 2006; Witkin et al., 2012; Barbosa et al., 2015). Therefore, lipid levels are important for the maintenance of nuclear shape. Importantly, LDs were recently discovered to be synthesized in the nucleoplasm and may contribute to NE maintenance by regulating DAG levels (Layerenza et al., 2013; Uzbekov and Roingeard, 2013; Cartwright et al., 2015; Grippa et al., 2015; Wolinski et al., 2015; Ohsaki et al., 2016; Romanauska and Köhler, 2018).

In mammalian hepatocyte lines, knockdown of SUN proteins increased nuclear lipid droplet formation, suggesting SUN proteins can influence membrane lipid composition and the DAG : PA balance (Ohsaki et al., 2016). In *S. cerevisiae, MPS3* can be completely deleted if the nuclear pore complex biogenesis factor *POM152* is also deleted (Rout et al., 2000; Fernandez-Martinez and Rout, 2009; Witkin et al., 2010). In this genetic background, deletion of *MPS3* showed an increase in DAG and ergosterol levels compared to the *pom152Δ* mutant alone (Friederichs et al., 2011), which is consistent with the increase in neutral lipids seen in mammalian hepatocytes. This same synthetic viable double *pom152Δ mps3Δ* mutant showed a more than two-fold increase in both TAG and phospholipid levels (Friederichs et al., 2011). Additionally, there is evidence that Mps3 promotes membrane rigidity. Overexpression of Mps3 at cold temperatures was found to be lethal and multiple *mps3* point mutations showed sensitivity to membrane fluidizing agents (Friederichs et al., 2011). Based on these observations, Mps3 mutants are likely to affect nuclear LD formation through changes in DAG and TAG levels. This would be an interesting avenue for future investigations.

A screen for point mutations in the ATP-binding P-loop of Mps3 created the lethal mutant Mps3-G186K (Friederichs et al., 2011). Galactose-induced expression of this mutant, integrated in the genome and in an otherwise wild type background, led to nuclear membrane expansion. Cells exhibited up to eight additional bilayers when the mutant was expressed, but not when the wild type was induced (Friederichs et al., 2011). The G186K mutation also exhibited a halt in mitotic progression suggestive of SPB duplication failure, which has been shown to cause nuclear flare formation elsewhere (Friederichs et al., 2011; Witkin et al., 2012). Interestingly, deletion of the acyl-coA synthetase, *FAA3,* rescued the Mps3-G186K phenotype (Friederichs et al., 2011). Faa3 prefers C16:0-C18:0 long-chain fatty acids, which are the most abundant saturated acyl tails found in yeast glycerophospholipids (Knoll et al., 1994; Grillitsch et al., 2011). Faa3 has also been identified as part of the yeast LD proteome (Grillitsch et al., 2011) and collaborates with the DAG acyltransferase Dga1 in the synthesis of TAG (Kamisaka et al., 2007). Interestingly, Dga1 was immuno‐affinity purified using antibodies directed towards Mps3‐FLAG protein (Bommi et al., 2019). Taken together, a model emerges whereby lipid metabolic enzymes and structural membrane proteins like Mps3 cooperate in lipid and NE homeostasis. Future work should aim to explore how the P-loop of Mps3 relates to membrane proliferation.

## MPS3 Links Lipid Metabolism and Telomere Organization at the Nuclear Periphery

Yeast telomeres are spatially organized within the nucleus: 32 telomeres in haploid cells cluster in 3 to 8 foci at the NE (Palladino et al., 1993). The nucleoplasmic N-terminal domain of Mps3 contains an acidic motif (75–150) that is important for tethering of telomeres in S-phase (Bupp et al., 2007). Consistently, ectopic expression of the N-terminal fragment (1–153) of Msp3, Mps3-N’, out-competes the N-terminus of endogenously expressed full-length Mps3 for binding to telomeres (Schober et al., 2009). The N-terminus of Mps3 physically interacts with the PAD domain of the silent information regulator protein Sir4 and this interaction is required for telomere tethering (Bupp et al., 2007). The Sir4 protein is essential for telomere clustering and anchoring across the cell cycle. In addition, Sir4 is important for the initiation of sub-telomeric transcriptional silencing by its direct interaction with double-stranded DNA binding protein Rap1 at telomeres and subsequent recruitment of additional SIR factors (Sir2 and Sir3) (reviewed in Grunstein and Gasser, 2013). The SIR complex nucleates along sub-telomeres leading to deacetylation of lysine residues on the tails of histone H3 and H4, repressing transcription (Hardy et al., 1992; Moretti et al., 1994; Wotton and Shore, 1997). The dispersion of SIR proteins from telomeres has been shown to induce transcriptional changes in the euchromatin (Taddei et al., 2009). Although telomere tethering per se is not required for transcriptional repression at telomeres (Mondoux et al., 2007), the pool of SIR factors concentrated at clustered telomeres, partly through Mps3, promotes telomere silencing (**Figure 2A**). Interestingly, and in line with the importance of Mps3-linked telomere tethering, *mps3*Δ75 to 150 shows telomere silencing defects (Bupp et al., 2007). In a second, Sir4-independent pathway, Mps3 interacts with telomere-bound yKu70/80 through Est1 (ever shorter telomeres) (Antoniacci et al., 2007), a non-catalytic subunit of telomerase. In this pathway, yKu80 interacts with Tlc1, the RNA template subunit of telomerase, and therefore, physically connects telomere regulation to Mps3 at the nuclear periphery (**Figure 2A**) (Schober et al., 2009). Importantly, Ikeda et al. showed that inhibition of sphingolipid synthesis by treatment with aureobasidin A or by disrupting *LCB1*, the enzyme which regulates the first committed step in sphingolipid synthesis, decreased telomere clustering. Using microarray analysis, the authors also showed that reducing sphingolipid synthesis by inhibiting inositol incorporation reduced expression of genes involved in telomere homeostasis, including Est1, Est2 and Est3 (Antoniacci et al., 2007; Ikeda et al., 2015). It is tempting to speculate that Mps3 is the intermediary between changes in sphingolipid levels and changes in telomere clustering.

Furthermore, and consistent with the S phase tethering function of Mps3, tethering of telomeres was decreased in the *mps3*-K-R mutant (Ghosh et al., 2012). This mutant has three lysine residues (K147, K148, and K150) mutated to arginine in the acid region of Mps3 (**Figure 1**), which are acetylated by the sister chromatid cohesion regulator Eco1 (Ghosh et al., 2012). The *mps3*-K-R mutant did not alter SPB duplication or Mps3 integration in the INM, but did disrupt nuclear morphology and the interaction of Mps3 with telomeres (Bupp et al., 2007; Ghosh et al., 2012). Consistently, deletion of the acidic motif of Mps3 had no effect on cell viability and showed no impact on spindle pole body (SPB) structure or organization (Bupp et al., 2007).

Acetylation of Mps3 by Eco1 may represent a point of regulation with lipid metabolic pathways, as lipid biosynthesis and degradation have been shown to alter nuclear acetyl-CoA pools impacting the epigenome (Berger and Sassone-Corsi, 2016; Etchegaray and Mostoslavsky, 2016; McDonnell et al., 2016; Su et al., 2016; van der Knaap and Verrijzer, 2016; Sivanand et al., 2018). Decreased fatty acid synthesis in yeast acetyl-CoA carboxylase mutants coincides with increased global histone acetylation levels (Galdieri and Vancura, 2012; Papsdorf and Brunet, 2019). The increase in histone H3 and H4 acetylation contributed to increased expression of genes known to be regulated by histone deacetylases (Galdieri and Vancura, 2012). Additionally, in cell cultures, acetyl-CoA derived from fatty acid breakdown was shown to account for 90% of the carbon source used in histone acetylation, directly upregulating genes involved in fatty acid metabolic processes (McDonnell et al., 2016). This strongly suggests a conserved mechanism of communication between the nucleus and lipid metabolism, which may be mediated by SUN proteins like Mps3.

Based on these connections, changes in NE composition could have downstream effects on gene expression regulation, particularly of non-essential genes silenced at sub-telomeres that are expressed upon environmental changes (Grunstein, 1997; Brown et al., 2010; Kueng et al., 2013). One function of Mps3 might involve organizing the genome, *via* telomere anchoring, such that the nucleus is poised for a transcriptional response through the cell cycle and under stress. Consistently, it was postulated that the repression of ribosomal protein genes in response to secretory stress is mediated by Mps3 (Mizuta and Warner, 1994; Mizuta et al., 1998; Yabuki et al., 2017).

## Genetic Interactions of MPS3

Based on the role of Mps3 in maintaining NE integrity and its role in telomere organization, we speculate that Mps3 serves to link NE membrane status to genome organization. Notably, telomere organization and telomere binding factors have genetic and physical interactions with factors regulating lipid homeostasis. For example, Est1 physically interacts with Lro1, a TAG synthesis enzyme (Lin et al., 2015). Lro1 was recently shown to localize to the INM under normal growth conditions and relocate in PA biosynthesis mutants, suggesting a physiological role for Lro1 in LD formation and in preservation of NE integrity through maintenance of DAG levels (Barbosa et al., 2019). Cells without *EST1* have short telomeres and positive genetic interactions with *pah1Δ* and negative interactions with loss of *OPI1* (Chang et al., 2011; Kyriakou et al., 2016). Conversely, cells lacking *RAP1* have longer telomeres and have negative genetic interactions with the loss of *LRO1* (Costanzo et al., 2016). The interactions between Est1 and PA metabolizing proteins warrant nuclear envelope studies in cells lacking *EST1*.

Lastly, to emphasize the functional interplay between telomere organization and lipid homeostasis, many of the proteins involved in lipid metabolism that have genetic interactions with Mps3 also show genetic interactions with factors at telomeres (**Figure 2B**). The Mps3–Y502H SUN domain mutant is synthetic sick with loss of *DEP1* and *NEM1*, two proteins involved in phospholipid biosynthesis. Loss of *DEP1* results in shorter telomeres and has negative interactions with the loss of *YKu80* (Costanzo et al., 2016), whereas loss of *NEM1* rescues end-protection defects in *cdc13-1*, a ts mutant for telomere specific single stranded binding factor that regulates telomerase (Addinall et al., 2008). The *mps3*-F592S mutation in the SUN domain is synthetic lethal with the loss of *SAC1*. Sac1 is a PI4P phosphatase that physically interacts with Scs2 (Manford et al., 2012). Moreover, it negatively interacts with telomere binding factors involved in silencing including yKu70 (Schuldiner et al., 2005; Addinall et al., 2011). The mps3-F592S variant is also synthetic lethal with loss of *APQ12*, an ER/NE integral membrane protein involved in lipid homeostasis and nuclear morphology, which itself has negative genetic interactions with loss of *SIR2* and *YKU70* (Friederichs et al., 2012). Finally, *mps3*-F592S also displayed synthetic growth defects with the deletion of *PSD1*. The Psd1 enzyme converts phosphatidylserine to phosphatidylethanolamine and its loss also displayed synthetic growth defects and lethality with deletions in *SAC1* and *EST1* respectively (Costanzo et al., 2010; Chang et al., 2011; Hoppins et al., 2011; Kuroda et al., 2011). Further characterization of these mutants will provide mechanistic insight into how Mps3’s “SUNny way” integrates lipid metabolic cues with nuclear envelope architecture and telomere association.

## Author Contributions

MLSP, SM-F, VZ, and JC all contributed to writing.

## Funding

MLSP is supported by a Queen Elizabeth II graduate award, SM-F holds a Alberta Cancer Board fellowship. VZ is supported by a Discovery grant and a Discovery Accelerator from NSERC. JC is supported by a Discovery grant from NSERC and an operating grant from CIHR.

## Conflict of Interest

The authors declare that the research was conducted in the absence of any commercial or financial relationships that could be construed as a potential conflict of interest.
